# Associations between VO_2max _and vitality in older workers: a cross-sectional study

**DOI:** 10.1186/1471-2458-10-684

**Published:** 2010-11-09

**Authors:** Jorien E Strijk, Karin I Proper, Linda Klaver, Allard J van der Beek, Willem van Mechelen

**Affiliations:** 1 Department of Public and Occupational Health, EMGO+ Institute for Health and Care Research, VU University Medical Center, van der Boechorststraat 7, 1081 BT Amsterdam, The Netherlands; 2 Body@Work, Research Center Physical Activity, Work and Health, TNO-VUmc, Amsterdam, The Netherlands

## Abstract

**Background:**

To prevent early exit from work, it is important to study which factors contribute to healthy ageing. One concept that is assumed to be closely related to, and therefore may influence healthy ageing, is vitality. Vitality consists of both a mental and a physical component, and is characterised by a perceived high energy level, decreased feelings of fatigue, and feeling fit. Since VO_2max_ gives an indication of one's aerobic fitness, which can be improved by increased levels of physical activity, and because feeling fit is one of the main characteristics of vitality, it is hypothesised that VO_2max_ is related to vitality. Therefore, the aim of this study was to investigate the associations between VO_2max_ and vitality.

**Methods:**

In 427 older workers (aged 45 + years) participating in the Vital@Work study, VO_2max_ was estimated at baseline using the 2-km UKK walk test. Vitality was measured by both the UWES Vitality Scale and the RAND-36 Vitality Scale. Associations were analysed using linear regression analyses.

**Results:**

The linear regression models, adjusted for age, showed a significant association between VO_2max_ and vitality measured with the RAND-36 Vitality Scale (β = 0.446; 95% CI: 0.220-0.673). There was no significant association between VO_2max_ and vitality measured with the UWES (β = -0.006; 95% CI:-0.017 - 0.006), after adjusting for age, gender and chronic disease status.

**Conclusions:**

VO_2max_ was associated with a general measure of vitality (measured with the RAND-36 Vitality Scale), but not with occupational health related vitality (measured with the UWES Vitality Scale). The idea that physical exercise can be used as an effective tool for improving vitality was supported in this study.

**Trial registration:**

NTR1240

## Background

Previous studies have shown that an age-related decline in health is a major contributor to early exit from work [1-3]. This leads to a shrinking number of economically active people and a future deficit of finances caused by the financial burden on both medical and social services [4]. Age-related decline in health is characterised by an increasing prevalence of chronic diseases, such as cardiovascular disease (CVD), diabetes mellitus (DM) and cancer, but also by the loss of body function (e.g. a decline in bone density mass, loss of renal tissue and a decline in lung function) [5]. To prevent early exit from work, it is thus important to promote and maintain good health.

One concept that is assumed to be closely related to, and therefore may influence health, is vitality. Vitality is related to both mental and physical factors of health [6-11]. regarding the mental factors, vitality reflects well-being, lower levels of fatigue, higher levels of emotional energy, mental resilience, and perseverance [6-10]. With respect to the physical factors, vitality is characterised by high energy levels and feeling "strong and fit" [10]. Vitality in the specific field of occupational health has been described by Schaufeli & Bakker (2003) as one of the three dimensions of work engagement and is characterised by "feeling full of energy, strong and fit, and being able to keep on working indefatigable" [10,11].

It is plausible to suggest that physical activity may improve both older workers' mental and physical components of vitality. As for the mental component of vitality, physical activity favourably affects mental health, well-being, and feelings of fatigue [12-15]. Furthermore, it has been shown that people who lead an active lifestyle are at reduced risk of suffering symptoms of depression [15]. As for the physical component of vitality, symptoms of physical illness, disability and immunological dysfunction have all been associated with a lower subjective vitality [7]. As described by Bouchard and colleagues (11), the positive effects of physical activity on health can be explained either through a direct relationship or an indirect one, namely through improved levels of health-related fitness, such as aerobic fitness.

Aerobic fitness is operationalised by VO_2max_, which is defined as the highest rate of oxygen consumption attainable during maximal or exhaustive exercise [16]. Several studies have reported an age-related decline in VO_2max _[17-19]. Vigorous physical activity can slow this age-related decline in VO_2max_. For physically active persons, the decline is approximately 5 percent per decade, while sedentary persons show a decline of 10 percent per decade [17]. Since VO_2max _gives an indication of one's aerobic fitness, which can be improved by increased levels of vigorous physical activity, and because feeling fit is one of the main characteristics of vitality, it is hypothesised that VO_2max _is associated to vitality. If VO_2max _is associated with vitality, a physical activity intervention can be considered as a promising tool to improve older workers' vitality. To date, the association between VO_2max _and vitality has not been studied among older workers. Therefore, the aim of this study was to investigate this association in older workers.

## Methods

### Study population

This study was conducted as part of the Vital@Work study, a Randomised Controlled Trial (RCT) evaluating a lifestyle intervention aimed at increasing (vigorous) physical activity levels in order to promote older workers' vitality [20]. Older workers (n = 730) were recruited from two major academic hospitals in the Netherlands. In order to be included, workers had to have a contract for at least 16 hours a week at the hospital. In addition, workers had to sign an informed consent form and had to indicate their risk for developing adverse health effects when becoming physically active. This risk was assessed by using the Physical Activity Readiness Questionnaire (PAR-Q) [21]. PAR-Q is composed of seven questions (yes/no) and has been designed to identify adults for whom physical activity might be inappropriate or those who should receive medical advice concerning the type of activity most suitable for them. Older workers who appeared to be at risk of for developing adverse health effects (one or more questions answered with 'yes') were excluded from the Vital@Work study. For the present study, older workers were excluded (n = 303) if they had not completed at baseline a 2-km UKK walk test. This study was approved by the Medical Ethics Committee of the VU university medical center. Of the 730 participants of the Vital@Work study, 427 workers completed a 2-km UKK walk test at baseline and were therefore included in this study.

### Measurements

#### Vitality

Vitality was measured by two vitality questionnaires: 1) the RAND-36 vitality scale [22] was used to measure vitality in general, and 2) the Utrecht Work Engagement Scale (UWES) vitality scale was used to measure vitality in the specific occupational setting of this study, namely older workers [10].

The RAND-36 Vitality Scale consist of four questions that refer to the past four weeks: 1) "Did you feel full of pep?" 2) "Did you have a lot of energy?" 3) "Did you feel worn out?" 4) "Did you feel tired?" The answers were rated on a six-point scale from "all of the time"(1) to "none of the time"(6) [22]. The RAND-36 vitality scale has shown to be sufficiently reliable; internal consistency was 0.82 (Cronbach's α) and a six-month test-retest stability coefficient was 0.63 [22]. The RAND-36 vitality score ranged from 0-100 points, calculated by (summing the points of each item - 4)/20) * 100. A higher score indicates a better subjective vitality.

The UWES is a 17-item questionnaire and is used to measure work engagement in the general working population [10]. The questionnaire consists of three scales, each measuring a component of work engagement, namely dedication, absorption, and vitality. Vitality is measured by six questions that refer to high levels of energy, fitness, resilience, the willingness to invest effort, not being easily fatigued, and persistence in the face of difficulties. The answers were rated on a 7-point scale from never (0) to daily (6). The UWES Vitality Score is calculated by the mean score of the six items. The UWES Vitality Scale has shown sufficient internal consistency (Cronbach's α = 0.82). Two longitudinal studies carried out in Australia and Norway showed one-year test-retest stability coefficients ranging between 0.64 and 0.71 [10].

#### VO_2max_

VO_2max _was estimated with the 2-km UKK walk test. This test has shown to be a feasible and accurate method for predicting VO_2max _in healthy 20-65 year old subjects [23,24]. The walk test was performed in a public park near the workplace. Before explaining the procedure of the test, workers were asked to: 1) fill out a form with their name, age and self-reported body height and body weight, and 2) put on a Polar heart rate monitor (type S610I; Polar Electro, Lake Success, NY). Subsequently, the procedure was explained in groups of on average 7 workers. Workers had to walk two kilometres individually at a pace as brisk as possible, but without running. At the finish, the heart rate and the performance time for the 2-km walk were noted by the research assistant. VO_2max _was estimated using gender-specific equations including age, body mass index (BMI), performance time for the walk (min) and heart rate at the end of the walk (HR). Data of self-reported body weight and body height were used to calculate BMI (kg/m^2^).

To calculate VO_2max _(ml × min^-1 ^× kg^-1^), the following regression equations were used [25]:

184.9 - 4.65 (time) - 0.22 (HR) - 0.26 (age) - 1.05 (BMI) for men,

116.2 - 2.98 (time) - 0.11 (HR) - 0.14 (age) - 0.39 (BMI) for women.

#### Covariates

Other variables relevant for this study were measured using a questionnaire and included age, gender, education (low = elementary school or less, medium = secondary education, and high = college/university), smoking (yes/no), and marital status (married/cohabitating/single/divorced/widowed). Information about chronic disease status (yes/no) was obtained using a 1-item question about chronic diseases from the Dutch Working Conditions Survey [26].

### Statistical analysis

Distributions of the continuous variables vitality, VO_2max _and age were described using means and standard deviations (SD); categorical variables were described using frequencies and percentages (Table 1). Correlation matrices were constructed to show the correlation between VO_2max_, and vitality measured by both the RAND-36 Vitality Scale and the UWES Vitality Scale (Table 2). To determine the association between VO_2max _and vitality, linear regression analyses were performed. Separate models were performed for the two different vitality measures (i.e. RAND-36 Vitality Scale and UWES Vitality Scale). Both crude and adjusted linear regression models were conducted (Table 3). Age, gender, education, marital status, smoking and having a chronic disease were included as potential confounders. Based on Twisk [27,28], a variable was classified as a confounder when the variable resulted in at least 10% change of the regression coefficient when included in the regression model. In addition, potential effect modification was assessed for all covariates, except for marital status, in order to investigate whether the association between VO_2max _and vitality is different for different subgroups (e.g. man versus women, younger workers versus older workers). This was assessed using interaction terms, which consisted of the independent variable and the covariate. Interaction terms were added separately to the analyses to determine their effects on the association between VO_2max _and vitality using a significance level p < 0.10. Statistical analysis were performed with the statistics software SPSS, version 15.0 (SPSS Inc. Chicago, Illinois, USA). The criterion p < 0.05 was applied to indicate statistical significance.

**Table 1:** Characteristics of subjects (n = 427)

Characteristics	N (%)
**Gender **(women)	306 (71.7%)

**Education**	
Low	40 (9.4%)
Middle	110 (25.8%)
High	277 (64.9%)

**Smoking **(yes)	39 (9.1%)

**Chronic diseases **(yes)	158 (37.0%)

**Marital Status**	
Married/cohabitating	318 (74.5%)
Single	72 (16.9%)
Divorced	30 (7.0%)
Widowed	7 (1.6%)

	**Mean ± SD**

**Age **(years)	52.4 ± 5.0

**VO**_**2max **_(ml × min^-1 ^× kg^-1^)	30.5 ± 7.0
Men	34.7 ± 8.3
women	28.8 ± 5.6

**UWES Vitality Scale**	4.9 ± 0.9

**RAND-36 Vitality Scale**	66.4 ± 16.9

**Table 2:** Correlation matrix for variables in regression models

	VO2max	Vitality UWES	Vitality RAND-36
**VO2max**	1	-0.068	0.163**

**Vitality UWES**		1	0.410**

**Vitality RAND-36**			1

** Correlation is significant at 0.001 level (2-tailed).

* Correlation is significant at 0.05 level (2-tailed)

**Table 3:** Linear regression analyses for VO_2max _and the RAND-36 Vitality Scale and the UWES Vitality Scale

		**VO**_**2max**_	
		
		B (95% CI)	p
**RAND-36 Vitality Scale**	Crude	0.395 (0.120-0.577)	0.003
	
	Adjusted*	0.446 (0.220-0.673)	0.000

**UWES Vitality Scale**	Crude	-0.007 (-0.018-0.003)	0.160
	
	Adjusted**	-0.006 (-0.017-0.006)	0.332

* adjusted for age

** adjusted for age, gender and chronic diseases

## Results

### Study population

The characteristics of the study population are summarised in table 1. The workers were, on average, 52.4 (SD = 5.0) years old and the majority of the population was highly educated (64.9%). Women represented 71.7% of the population and the majority of the workers was married/cohabitating (74.5%). The mean VO_2max _was 34.7 (SD = 8.3) ml × min^-1 ^× kg^-1 ^for men and 28.8 (SD = 5.6) ml × min^-1 ^× kg^-1 ^for women, which represents average levels of VO_2max _for both gender groups [29]. Workers had a mean score of 4.9 (SD = 0.9) on the UWES vitality scale, which correspondents with the category 'high' according to the UWES classification [10,11]. The mean score on the RAND-36 vitality scale was 66.4 (SD = 16.9), which correspondents with the average norm score of this scale [22].

### Correlations VO_2max _and vitality (RAND-36 & UWES)

The correlations between *VO*_*2max *_and the two measurements of vitality are presented in Table 2. There was a positive correlation between VO_2max _and vitality measured by the RAND-36 Vitality Scale (r = 0.16, p < 0.001). There was no significant correlation between VO_2max _and vitality measured by the UWES Vitality Scale (r = -0.07, p < 0.160). Finally, the two vitality scales (i.e. RAND-36 Vitality Scale and UWES Vitality Scale) were positively correlated (r = 0.41, p < 0.001).

### Associations between VO_2max _and the RAND-36 Vitality Scale

Results of crude and adjusted linear regression analyses for the association between VO_2max _and the RAND-36 Vitality Scale are presented in table 2. Crude analysis showed that each point increase of VO_2max _was associated with a significant increase of 0.395 points on the RAND-36 Vitality Scale (β: 0.395, 95% CI: 0.120-0.577, p < 0.003). After adjusting for the potential confounder age, the association between VO_2max _and vitality measured by the RAND-36 Vitality Scale became significantly stronger (β: 0.446, 95% CI: 0.220-0.673, p < 0.000).

### Associations between VO_2max _and the UWES Vitality score

Table 2 also presents the results of the crude and adjusted linear regression analyses for the association between VO_2max _and the UWES Vitality scale. Crude analysis showed that there was no significant association between VO_2max _and vitality measured with the UWES Vitality Scale (: -0.007, 95% CI: -0.018-0.00, p < 0.160). Age, gender, and chronic diseases appeared to be confounders in this association since these variables caused a more than 10% change of the regression coefficient after adding to the regression model. After adjustment for these confounders, there was still no association between VO_2max _and vitality measured by the UWES Vitality Scale (: -0.006, 95% CI: -0.017-0.006, p < 0.332).

## Discussion

The aim of this study was to investigate the association between VO_2max _and vitality in older workers. This study showed a positive and significant association between VO_2max _and vitality measured by the RAND-36 Vitality Scale. However, there was no association between VO_2max _and vitality measured by the UWES Vitality Scale.

Our findings concerning the RAND-36 vitality scale were indirectly supported by a recent cross-sectional Finnish study, which showed that a higher cardiorespiratory fitness (CRF), expressed as a Physical Fitness Index (PFI) based on VO_2max _and muscle strength, was associated with a higher vitality measured with the RAND-36 Vitality Scale [30]. Results from another study of middle-aged male workers showed that there was no correlation between VO_2max _and the RAND-36 Vitality Scale [31]. The gender focus and the small study sample (n = 73) may partly account for the difference in results of this and the present study. Besides the direct relationship between VO_2max _and vitality, there is scientific evidence for the relationship between physical activity and vitality. The review of Puetz [13] demonstrated considerable evidence between physical activity and a 41% reduced risk of experiencing low energy levels and fatigue measured with the RAND-36 Vitality Scale, when active adults were compared with sedentary peers. Since vitality can be defined as a component of health-related quality of life (HRQoL), the RAND-36 is a questionnaire to assess HRQoL. There has been a recent study investigating the association between CRF and HRQol. In this observational study of healthy United States navy men, relatively higher levels of CRF (expressed as maximal MET level, which was calculated from sub maximal VO_2max_), were associated with higher levels of HRQoL [32]. As for the UWES vitality scale, there have not been any published studies investigating the association between VO_2max _and the UWES vitality scale or the total concept of work engagement, respectively.

### Methodological considerations

In this study, VO_2max _was measured using the UKK walk test, which provides an indirect measure of VO_2max_. The optimal way for measuring VO_2max _is by a maximal exercise test (i.e. treadmill test). However, considering the large target population, the UKK walk test was most practical, suitable and socially acceptable [25]. Moreover, research has shown that the VO_2max _calculated by the UKK walk test predicted 73-75% of the variance in VO_2max _[25]. Furthermore, although measuring body height and body weight are quick and easy measures, for practical reasons self-reported body height and body weight were in this study used to calculate BMI, and subsequently VO_2max_. These self-reported measures may have been biased because body weight is often under-reported, while body height is often over-reported [33-35]. Nevertheless, several studies have shown that self-reported BMI is reasonably accurate [36-38]. Another consideration is that this study investigated the associations between VO_2max _and vitality using a cross-sectional design, from which we cannot determine a direct cause and effect relationship. Also, generalisibility of this study may be limited because it was conducted only among hospital workers aged 45 years and older. Future longitudinal research among a general working population is needed to provide a better understanding about this direct cause and effect relationship.

### Measuring two constructs of vitality

This study showed that the correlation between the RAND-36 Vitality Scale and the UWES Vitality Scale was moderate (r = 0.41, p < 0.001). When two scales measure the same construct, a higher correlation between the two scales can be expected. Therefore, it can be assumed that the two vitality scales measure two different constructs of vitality, namely a physical and a mental component, respectively. Considering the origin of both the vitality measurements, this assumption seems plausible.

The RAND-36 is the Dutch version of the MOS 36-item Short-form Health Survey (SF-36) [39], which was designed for use in clinical practice and research, health policy evaluations, and general population surveys. The RAND-36 includes one multi-item scale that assesses 8 health concepts, including vitality [22]. As described in the methods, the RAND-36 Vitality Scale consists of questions referring to perceived energy level and fatigue [9]. This may indicate that the RAND-36 Vitality Scale represents mainly the physical component of vitality.

The UWES on the other hand, has been developed by Schaufeli and Bakker who were also involved in the development of the Utrecht BurnOut Scale (UBOS) for measuring burnout, which is work-related psychological exhaustion [40]. The UWES was developed by reversing the three negative dimensions of the UBOS (i.e. exhaustion, cynicism, and professional efficacy) into the three positive dimensions of the UWES (i.e. vitality, dedication, and absorption) [40,41]. Considering the origin of the UWES, it is plausible that the UWES Vitality Scale focuses mainly on the mental component of vitality. For the evaluation of the effectiveness of future preventive (occupational) vitality programs, it is essential to have the availability of a reliable and valid questionnaire that covers the entire concept of vitality. Since vitality seems to consist of a mental as well as a physical component, the findings of our study imply that neither the RAND-36 Vitality Scale nor the UWES Vitality Scale covers the entire concept of vitality. Therefore, for future research it is recommended to be focussed on the development and evaluation of such a questionnaire.

## Conclusions

This study showed a positive and significant association between VO_2max _and general vitality measured by the RAND-36 Vitality Scale. However, there was no significant association between VO_2max _and vitality measured by the occupational health specific UWES Vitality Scale. The idea that physical exercise can be utilised as an effective tool for improving vitality was supported in this study, since an improvement in VO_2max _was associated with an increased vitality (RAND-36). This will be further investigated among older workers in the Vital@Work study [20].

## Competing interests

The authors declare that they have no competing interests.

## Authors' contributions

JES, KIP, AvdB, and WvM provided support in the design of the Vital@Work study. JES coordinated the data collection, performed data analysis and drafted the manuscript. LK performed data collection, analysed the first data and contributed to the design of the manuscript. KIP, AvdB, and WvM contributed intellectual input and provided support for this study. All authors contributed to the further writing of the manuscript. All authors have read and corrected draft versions of the manuscript and approved the final manuscript.

## Pre-publication history

The pre-publication history for this paper can be accessed here:

http://www.biomedcentral.com/1471-2458/10/684/prepub

## References

[B1] Cai L Kalb G Health status and labour force participation: evidence from Australia Health Econ 2006 15 241 261 10.1002/hec.1053 16229055

[B2] Lund T Iversen L Poulsen KB Work environment factors, health, lifestyle and marital status as predictors of job change and early retirement in physically heavy occupations Am J Ind Med 2001 40 161 169 10.1002/ajim.1084 11494344

[B3] Schuring M Burdorf L Kunst A Mackenbach J The effects of ill health on entering and maintaining paid employment: evidence in European countries J Epidemiol Community Health 2007 61 597 604 10.1136/jech.2006.047456 17568051 PMC2465755

[B4] Minicuci N Marzari C Maggi S Noale M Senesi A Crepaldi G Predictors of transitions in vitality: the italian longitudinal study on aging J Gerontol A Biol Sci Med Sci 2005 60 566 573 15972603 10.1093/gerona/60.5.566

[B5] Young A Ageing and physiological functions Philos Trans R Soc Lond B Biol Sci 1997 352 1837 1843 10.1098/rstb.1997.0169 9460068 PMC1692134

[B6] Shirom A Bakker A, Leiter M Feeling energetic at work: On vigor's antecedents Work Engagement: recent developments in theory and research 2010 New York: Psychology Press 69 84

[B7] Ryan RM Frederick C On energy, personality, and health: subjective vitality as a dynamic reflection of well-being J Pers 1997 65 529 565 10.1111/j.1467-6494.1997.tb00326.x 9327588

[B8] McNair DM Lorr M Droppleman LF Manual for the profile of mood states 1971 San Diego: Educational and Industrial Testing Service

[B9] McHorney CA Ware JE Jr Raczek AE The MOS 36-Item Short-Form Health Survey (SF-36): II. Psychometric and clinical tests of validity in measuring physical and mental health constructs Med Care 1993 31 247 263 10.1097/00005650-199303000-00006 8450681

[B10] Schaufeli WB Bakker AB Utrecht Work Engagement Scale Occupational Health Psychology Unit Utrecht University 2003

[B11] Schaufeli WB Bakker AB Bevlogenheid: een begrip gemeten Gedrag en Organisatie 2004 17 90 112

[B12] Penedo FJ Dahn JR Exercise and well-being: a review of mental and physical health benefits associated with physical activity Curr Opin Psychiatry 2005 18 189 193 10.1097/00001504-200503000-00013 16639173

[B13] Puetz TW Physical activity and feelings of energy and fatigue: epidemiological evidence Sports Med 2006 36 767 780 10.2165/00007256-200636090-00004 16937952

[B14] World Health Organisation The World Health Report, reducing Risks, promoting Healthy Life 2002 Geneva, Switzerland, World Health Organisation (WHO) Ref Type: Report

[B15] World Health Organisation (WHO) Promoting Mental Health. Concepts, Emerging Evidence, Practice. 2-15 2005 Geneva, Switzerland, World Health Organisation (WHO) Ref Type: Report

[B16] Wilmore JH Costill DL Kenney WL Energy Expenditure and Fatigue Physiology of Sport and Exercise 2008 100 118

[B17] Bouchard C Shephard R Stephens T Physical activity and fitness with age among sex and ethnic groupsphysical activity and health 1994 38 47

[B18] Ilmarinen JE Aging workers Occup Environ Med 2001 58 546 552 10.1136/oem.58.8.546 11452053 PMC1740170

[B19] Betik AC Hepple RT Determinants of VO2 max decline with aging: an integrated perspective Appl Physiol Nutr Metab 2008 33 130 140 10.1139/H07-174 18347663

[B20] Strijk JE Proper KI van der Beek AJ van MW The Vital@Work Study. The systematic development of a lifestyle intervention to improve older workers' vitality and the design of a randomised controlled trial evaluating this intervention BMC Public Health 2009 9 408 10.1186/1471-2458-9-408 19903345 PMC2777874

[B21] Shephard RJ PAR-Q, Canadian Home Fitness Test and exercise screening alternatives Sports Med 1988 5 185 195 10.2165/00007256-198805030-00005 3368685

[B22] van der Zee KI Sanderman R Het meten van gezondheidstoestand met de RAND-36: een handleiding Groningen: Noordelijk Centrum voor Gezondheidsvraagstukken 1993

[B23] Raija MT Laukkanen RMT Oja P Ojala ME Vuori IM Feasibility of a 2-km walking test for fitnes assessment in a population study Scand J Soc Med 1992 20 119 125 1496331 10.1177/140349489202000210

[B24] Oja P Laukkanen R Pasanen M Tyry T Vuori I A 2-km walking test for assessing the cardiorespiratory fitness of healthy adults Int J Sports Med 1991 12 356 362 10.1055/s-2007-1024694 1917218

[B25] Oja P Mänttäri A Pokki T Kukkonen-Harjula K Laukkanen RMT Malmberg J UKK Walk Test - Tester's guide 2001 Tampere, Finland: UKK Institute

[B26] TNO. De nationale enquete arbeidsomstandigheden (NEA) 2008 Ref Type: Report

[B27] Twisk JWR Applied Multilevel Analysis 2006 Cambridge: Cambridge University Press

[B28] Twisk JWR Introductie in de Biostatistiek [Introduction into Biostatistics] 2007 Maarsen: Elsevier Gezondheidszorg

[B29] American College of Sports Medicine ACSM's Guideline for Exercise Testing and Prescription 2006 Seventh Baltimore, USA, Lippincott Williams & Wilkins Ref Type: Report

[B30] Hakkinen A Rinne M Vasankari T Santtila M Hakkinen K Kyrolainen H Association of physical fitness with health-related quality of life in Finnish young men Health Qual Life Outcomes 2010 8 15 10.1186/1477-7525-8-15 20109241 PMC2835678

[B31] Sorensen L Honkalehto S Kallinen M Pekkonen M Louhevaara V Smolander J Are cardiorespiratory fitness and walking performance associated with self-reported quality of life and work ability? Int J Occup Med Environ Health 2007 20 257 264 10.2478/v10001-007-0023-3 17932015

[B32] Sloan RA Sawada SS Martin CK Church T Blair SN Associations between cardiorespiratory fitness and health-related quality of life Health Qual Life Outcomes 2009 7 47 10.1186/1477-7525-7-47 19476640 PMC2695434

[B33] Gorber SC Tremblay M Moher D Gorber B A comparison of direct vs. self-report measures for assessing height, weight and body mass index: a systematic review Obes Rev 2007 8 307 326 10.1111/j.1467-789X.2007.00347.x 17578381

[B34] Spencer EA Appleby PN Davey GK Key TJ Validity of self-reported height and weight in 4808 EPIC-Oxford participants Public Health Nutr 2002 5 561 565 10.1079/PHN2001322 12186665

[B35] Niedhammer I Bugel I Bonenfant S Goldberg M Leclerc A Validity of self-reported weight and height in the French GAZEL cohort Int J Obes Relat Metab Disord 2000 24 1111 1118 10.1038/sj.ijo.0801375 11033979

[B36] Craig BM Adams AK Accuracy of body mass index categories based on self-reported height and weight among women in the United States Matern Child Health J 2009 13 489 496 10.1007/s10995-008-0384-7 18607705 PMC2731685

[B37] Dekkers JC van Wier MF Hendriksen IJ Twisk JW van MW Accuracy of self-reported body weight, height and waist circumference in a Dutch overweight working population BMC Med Res Methodol 2008 8 69 10.1186/1471-2288-8-69 18957077 PMC2605752

[B38] Sherry B Jefferds ME Grummer-Strawn LM Accuracy of adolescent self-report of height and weight in assessing overweight status: a literature review Arch Pediatr Adolesc Med 2007 161 1154 1161 10.1001/archpedi.161.12.1154 18056560

[B39] Ware JE Jr Sherbourne CD The MOS 36-item short-form health survey (SF-36). I. Conceptual framework and item selection Med Care 1992 30 473 483 10.1097/00005650-199206000-00002 1593914

[B40] Schaufeli W van Dierendonck D UBOS -- Utrechtse Burnout Schaal. Handleiding. [The Utrecht Burnout Scale (UBOS)] De Psycholoog 2001 9 12

[B41] Schaufeli W Salanova M González-Romá V Bakker A The measurement of engagement and burnout: A two sample confirmatory factor analytic approach Journal of Happiness Studies 2002 71 92 10.1023/A:1015630930326

